# Unexpected Tension Pneumothorax-Hemothorax during Induction of General Anaesthesia

**DOI:** 10.1155/2019/5017082

**Published:** 2019-02-24

**Authors:** Ekaterini Amaniti, Chrysoula Provitsaki, Panagiota Papakonstantinou, George Tagarakis, Konstantinos Sapalidis, Ioannis Dalakakis, Dimitrios Gkinas, Vasilios Grosomanidis

**Affiliations:** ^1^Aristotle University of Thessaloniki, Stilponos Kyriakidi 1 Thessaloniki 54636, Greece; ^2^AHEPA University Hospital, Aristotle University of Thessaloniki, Stilponos Kyriakidi 1 Thessaloniki 54636, Greece

## Abstract

Tension pneumothorax during general anaesthesia is a rare but possibly deleterious event, especially where predisposing factors are absent or unknown, making diagnosis even challenging. We describe a case of a healthy middle-aged woman, who was planned to receive general anaesthesia for total thyroidectomy. After intubation, the patient experienced marked hypoxemia (SpO2=75%), hypotension, and tachycardia. Manual positive pressure ventilation seemed to worsen hypoxemia and tachycardia, while apnoeic oxygenation through circle system with valve open slightly improved cardiorespiratory collapse. The effect of positive ventilation, along with the absence of breath sounds in the right hemithorax and cardiorespiratory collapse, established the diagnosis of tension pneumothorax, managed immediately with emergency thoracentesis and placement of a thoracostomy tube. The patient was improved and pneumothorax was confirmed with chest X-ray and CT. The latter also confirmed the presence of bilateral multiple bullae. The operation was postponed and the patient was extubated a few hours later, in good condition. After thorough evaluation for any systemic disease, which was negative, the patient underwent two-stage thoracotomy for bullectomy.

## 1. Introduction

Tension pneumothorax during general anaesthesia is a rare but deleterious event in the perioperative setting, which may cause severe cardiorespiratory collapse, leading to brain damage or even death [1]. Specific operations in proximity to parietal pleura, laparoscopy, or application of regional block or central venous cannulation are among interventions, where a pleural tear is quite possible, enabling iatrogenic pneumothorax formation [2, 3], while positive pressure ventilation is the most common aetiology of pneumothorax in ICU mechanically ventilated patients [4]. Under these conditions, the development of tension pneumothorax is a well-established phenomenon, necessitating fast and proper diagnosis and rapid drainage [4].

We describe a case of a healthy middle-aged woman, who was planned to receive general anaesthesia for total thyroidectomy. Despite the absence of any surgical or anaesthesia-related factor for pneumothorax formation, the patient experienced intraoperative tension pneumothorax. After initial resuscitation and pneumothorax drainage, the patient was found to suffer from multiple, undiagnosed emphysematous bullae. Thyroidectomy was postponed and rescheduled one year later, after two-stage thoracotomy for bilateral bullectomy.

## 2. Case Presentation

Female patient, aged 57 years, BMI=23, was planned to undergo elective thyroidectomy, due to multinodular goiter. Her medical history included arterial hypertension, controlled with beta blocker and angiotensin receptors II antagonist. The patient has satisfactory physical status with no symptoms from cardiac and respiratory systems (no dyspnoea or coughing, no smoking history, asthma or COPD, >4 METS). No other concerns were raised regarding her family or previous anaesthetic history (she had received general anaesthesia 10 years ago for appendectomy without complications). Physical examination and laboratory testing did not reveal any findings and the patient had no prognostic factors of difficult airway management (Mallampati classification: II, thyromental distance > 6 cm, interincisional gap> 4 cm, and cervical spine extension > 90°). Chest X-ray was normal, regarding lung parenchyma but with slightly enlarged cardiac silhouette. This finding, in conjunction with Q waves, seen on ECG, made further cardiologic consultation necessary. However, no signs of cardiac disease were revealed, either by clinical examination, or by transthoracic ultrasound performed. The patient was scheduled for the operation a few days later and discharged home with the appropriate recommendations for medications and preoperative fasting.

On the day of operation, after establishment of the basic monitoring and intravenous line, anaesthesia was induced with midazolam 2 mg, Fentanyl 3 *μ*g/Kg, and Propofol 2.5 mg/Kg and muscle relaxation was achieved with Cisatracurium 0.2 mg/Kg. Mask ventilation was difficult but pretty adequate. During first attempt of intubation, direct laryngoscopy resulted in a Cormarck=Lehane III and despite the use of elastic bougie was unsuccessful without passing the vocal cords. An LMA # 4 was inserted and a more experienced anaesthetist was called. At that time SpO_2_ was dropped to 95% with FiO_2_=1 with controlled ventilation with MV= 3.5 L/Min and adequate capnometry. After several minutes of ventilation with LMA and as SpO_2_ was 99% intubation was reattempted with the use of C-mac videolaryngoscope (Karl Stortz® Tuttlingen, Germany) and an elastic bougie. Second attempt was successful, as laryngoscopic view was significantly improved (Cormarck-Lehane: IIa), without any suspicion of airway trauma. A 7.5 mm neural integrity monitor (NIM) electromyogram (EMG) tracheal tube (Comepa®, Bagnolet, France) was used for intubation. Attention was paid for the proper placement of the tube (the silver color-coded contact band placed between the vocal cords). Anaesthesia was then maintained with desflurane 1 MAC in air/oxygen with FiO_2_=0.5.

**Figure 1:**
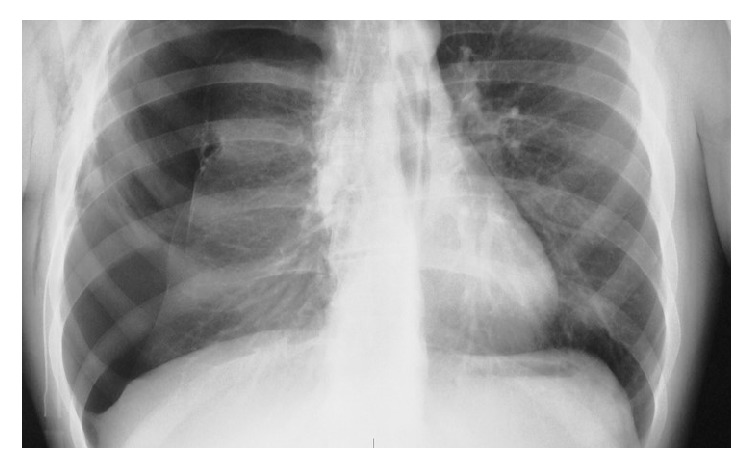
Operating theater chest X-ray with right pneumothorax.

**Figure 2:**
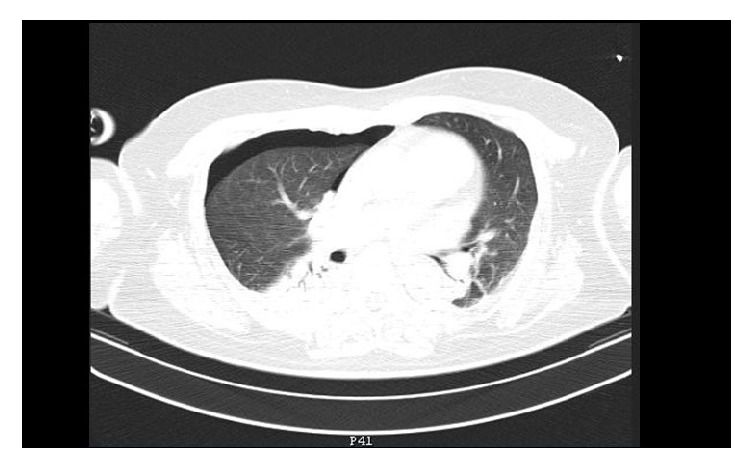
CT in the thorax showing right hemothorax and pneumothorax.

**Figure 3:**
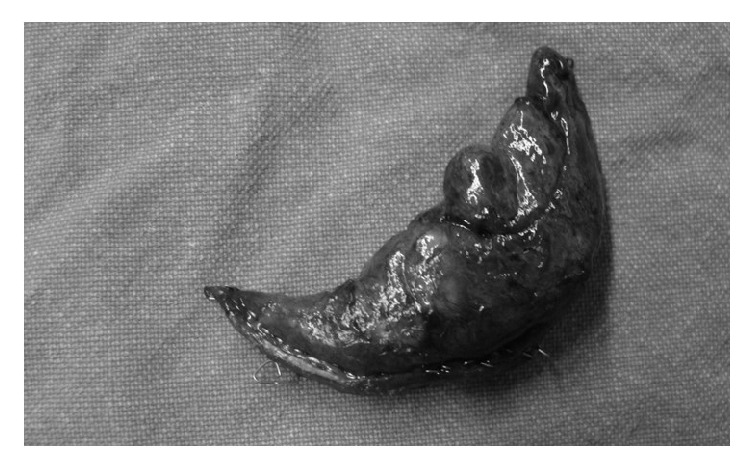
Resected bullae.

Despite adequate ventilation (MV=5 L/min, Vt=480, RR=11, PEEP=3, and EtCO_2_=38mmHg) a sudden drop in SpO_2_ was observed during the next 2 minutes after intubation. SpO_2_ dropped at 75% with FiO_2_=1, with a concomitant hypotension (75/40 mmHg) and tachycardia (95bpm). Respiratory monitoring showed only slightly elevated Peak Inspiratory Pressure (28cmΗ_2_O). Since this incident happened before commencement of the operation, surgical drapes were removed and the patient was manually ventilated. During manual ventilation, it was observed that positive ventilation led to greater SpO_2_ drop, while stopping ventilation with open airway pressure release valve and FiO_2_=1 (apnoeic oxygenation), resulting in improvement of SpO_2_ (85-87%). Chest auscultation revealed excessively reduced sounds in the right hemithorax. Based on these clinical findings, the suspicion of tension pneumothorax was raised. A thoracic surgeon was immediately called, along with bed-side X-ray. The thoracic surgeon performed emergency insertion of a 14G venous cannula to the 2nd intercostal space at midclavicular line with immediate drainage of bubbles and haemorrhagic fluid and improvement of hemodynamics (BP: 95 mmHg, HR: 88 bpm) and SpO2: 92%. Chest X-ray confirmed the diagnosis of pneumothorax (Figure 1). A thoracostomy tube was inserted with further improvement of oxygenation and drainage of 100 ml haemorrhagic fluid. The operation was postponed and the patient was transferred to the CT-suite, under sedation and controlled ventilation. CT scan revealed haemothorax and pneumothorax to the right hemithorax (Figure 2), perihepatic fluid collection, and fluid in the Morison's pouch. Moreover, the diagnosis of multiple bullae was posed. The patient returned to the Postanesthesia Care Unit under sedation. ABG analysis was satisfactory (pH=7.35, PO_2_=123 mmHg with FiO_2_=0.5, and pCO_2_=38 mmHg). Hence, recovery from sedation and extubation was decided, which was uneventful. The patient was discharged a few days later. After investigation for pulmonary cystic disease, the patient underwent two stages of bilateral thoracotomy for partial parietal pleurectomy, lung apicectomy, and bullectomy, during the next few months (Figure 3). One year later she underwent total thyroidectomy for multinodular goiter without any perioperative complications.

## 3. Discussion

Pneumothorax during general anesthesia is a relatively rare but severe complication, which may lead to adverse events. In an analysis of 2046 closed claims regarding adverse respiratory events, pneumothorax occurred in 3% of cases [1]. More recent closed claims analyses showed similar results [5]. Furthermore, pneumothoraces due to barotrauma or airway manipulation were associated with poor outcome such as death or serious brain injury [1]. However, the absence of any predisposing factor, like vigorous airway instrumentation, ventilator malfunction, or central venous catheterization, made the diagnosis more challenging regarding acute profound intraoperative hypoxemia.

During preoperative evaluation, the patient was considered pretty healthy, with no history of smoking, dyspnea, coughing, or any other respiratory complaints. Preoperative chest X-ray was rather normal regarding lung parenchyma. This is in accordance with current knowledge of inability of chest X-ray to show bullae. As the patient was not known to have lung disease or bullae, routine practice was followed and the patient was ventilated with 8 ml/kg,” RR of 10/min, and PEEP=4 cmH2O.

Previous studies have shown that pneumothorax seemed to be more related to tidal volumes than peak inspiratory pressures [6]. Nevertheless, a recent meta-analysis showed that the effects of low volume ventilation (6-8.1 ml/Kg), compared to high volume (10-12ml/Kg) on pneumothorax, were uncertain [6]. On the other hand, the effect of PEEP is more complex. Higher levels of PEEP have been observed in patients with pneumothorax during ICU stay, but this could probably be attributed to the high incidence of ARDS and not PEEP per se [7]. However, it should be noted that the presence of emphysematous bullae is a quite different clinical scenario. A recent study showed that, during VAT surgery, the bullae were expanded enough to be identified as “bullae” due to the sufficient pressure inside the bullae even when the normal lung parenchyma was maintained at approximately 30-50% of the expansion level under low pressure ventilation by means of HFJV + PEEP [8]. Current practice recommends the avoidance of positive pressure ventilation in the presence of bullae [9]. In this case, mechanical ventilation is considered the main predisposing factor for rupture of bullae and hemopneumothorax formation, which in the presence of mechanical ventilation caused the development of tension pneumothorax.

During the first few minutes of oxygenation deterioration and hemodynamic collapse and before drapes were removed, diagnosis of pneumothorax was impossible. Nevertheless, the presence of high peak and plateau pressure made anaesthetic team switches to manual ventilation. The observation that vigorous positive pressure inflations further exacerbated hypoxemia, while disconnection from the circle system led to improvements of oxygenation, was a rather empirical clinical sign that positive ventilation had a negative impact on oxygenation. Two possible explanations were considered: the development of acute severe bronchospasm with air-trapping or tension pneumothorax. Both situations are negatively impacted by positive ventilation and have similar hemodynamic and respiratory consequences, while at the same time, extreme air-trapping can also lead to barotrauma [10, 11]. While diagnosis of air-trapping and auto-PEEP is considerably difficult in the operating theater, unilateral absence of respiratory sounds in the right hemithorax supported the clinical diagnosis of pneumothorax [12].

The clinical diagnosis of pneumothorax was mainly supported by the cardiorespiratory collapse, in conjunction with the absence of breath sounds in the right hemithorax. However, in cases of left pneumothorax, clinical diagnosis is more difficult, as absence of breath sounds might be misinterpreted as unintended right bronchial intubation. In this scenario, high inspiratory peak pressures and plateau pressures are also present. If signs of circulatory compromise are slight and other indirect signs like jugular vein enlargement remain unnoticed, the diagnosis could be set with great delay. Another point of concern is the use of special EMG monitoring endotracheal tube. This tube is carefully inserted to an appropriate depth, in order to achieve optimum contact with vocal cords. While no differences in depth insertion have been shown regarding height and weight, the possibility of bronchial intubation due to anatomical reasons could not be excluded [13]

Although ventilator settings were acceptable (Vt=8 ml/kg), the possibility or bullae rupture could not be excluded. However, it is possible that bullae rupture occurred during the phase of manual ventilation. The subjective sense of difficult manual ventilation urged anaesthetist to close the adjustable pressure release valve up to 30 cm H_2_O. Manual ventilation has been found to induce increased inspiratory peak flow rates and higher peak airway pressures, compared to controlled pressure ventilation [14]. The rupture of bullae during manual ventilation is further supported by the rapid cardiorespiratory deterioration after intubation and start of controlled ventilation. It is not known which proportion of Peak inspiratory pressure and flow is transmitted to the bullae. Nevertheless, the establishment of a continuous positive end-expiratory pressure, along with the behaviour of bullae like a paper bag, may contribute to the development of pneumothorax [15].

In conclusion, although rare, the possibility of intraoperative pneumothorax formation should always be considered in cases of unexpected cardiorespiratory collapse, even in cases where no predisposing factors are present. Emphysematous bullae could exist in absence of clinical symptoms and signs or radiographic findings. Fast clinical diagnosis, along with urgent thoracentesis, could be a salvage manoeuvre, especially under positive ventilation.
